# A Wearable and Highly Sensitive PVDF–TrFE–BaTiO_3_ Piezoelectric Sensor for Wireless Monitoring of Arterial Signal

**DOI:** 10.1021/acsaelm.5c00548

**Published:** 2025-07-14

**Authors:** Qinrong He, Huxi Wang, Jungang Zhang, Negin Ghahremani Arekhloo, Xenofon Karagiorgis, Bhavani Prasad Yalagala, Peter J. Skabara, Hadi Heidari, Dagou A. Zeze, Ensieh S. Hosseini

**Affiliations:** † Department of Engineering, 3057 Durham University, DH1 3LE Durham, U.K.; ‡ Key Laboratory of Bionic Engineering (Ministry of Education), College of Biological and Agricultural Engineering, 12510 Jilin University, Changchun, Jilin 130022, China; § James Watt School of Engineering, 3526 University of Glasgow, G12 8QQ Glasgow, U.K.; ∥ School of Chemistry, University of Glasgow, G12 8QQ Glasgow, U.K.

**Keywords:** PVDF–TrFE/BaTiO_3_, electrospinning, piezoelectric sensor, pulse monitoring, wireless data transfer

## Abstract

As wearable electronics advance, there is a growing need for flexible sensors with high sensitivity to detect even the slightest mechanical stimuli for real-time monitoring across various applications. This study presents a poly­(vinylidene fluoride-*co*-trifluoroethylene) (PVDF–TrFE)-based flexible piezoelectric sensor, developed by electrospinning a composite of PVDF–TrFE and barium titanate (BaTiO_3_). The PVDF–TrFE with 3 wt % BaTiO_3_, referred to as PVDF–TrFE (3 wt % BTO), exhibits higher crystallinity, increased β-phase content, and enhanced piezoelectric response, achieving a pressure sensitivity of 0.37 V/kPa within a pressure range of 6.4–16 kPa at a fixed frequency of 7 Hz. The flexible sensor developed is also characterized by its ability to detect lower pressure ranges with a linear pressure sensitivity of 0.18 V/kPa over a range of 6.4–22.4 kPa at a fixed frequency of 2 Hz. It also exhibits a frequency sensitivity of 0.7 V/Hz within a frequency range of 2–5 Hz at a constant pressure of 6.4 kPa. The fabricated sensors were integrated with a microcontroller and wireless data transfer system to form a wearable sensor patch that detects biomechanical signals such as wrist bending and radial artery pulse signals, ensuring reliable monitoring of biomechanical signals. Furthermore, spatially sensitive detection was achieved by creating a 3 × 3 pressure array sensor to pinpoint pressure locations. With the wireless data transfer system, sensor signals can be sent to a smartphone, which acts as a pressure locator to track external force positions. This work demonstrates that the pressure sensing device developed using the PVDF–TrFE (3 wt % BTO) sensor has significant and promising potential for real-time physiological detection and wearable healthcare monitoring.

## Graphical abstract

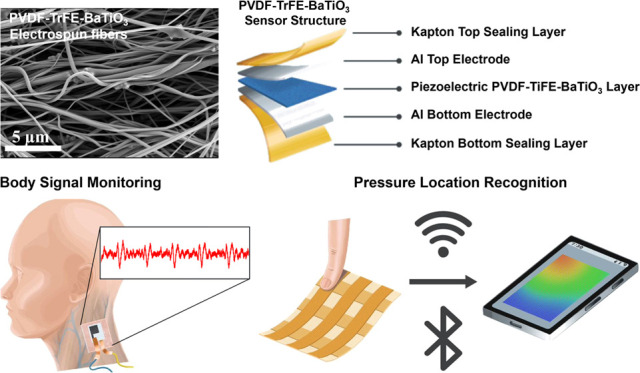

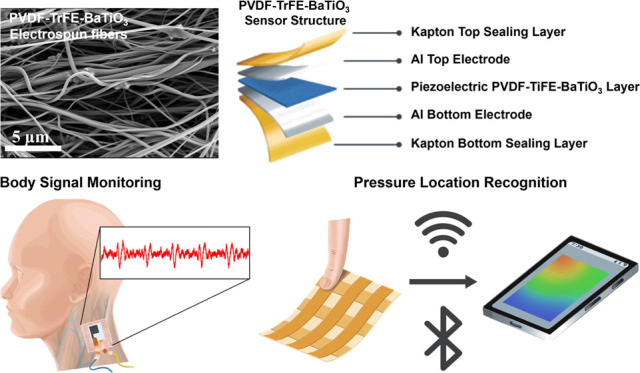

## 1 Introduction

As the internet of things (IoT) advances, there is a growing demand for portable, comfortable, and flexible sensors for real-time detection of subtle movements and physiological signals in sports and health diagnostics.^1–4^ For example, continuous monitoring of body movements and arterial pulse signals can provide valuable insights into personal health and enable therapeutic interventions. Wearable sensors, in particular, are expected to conform to the complex contours of the human body, making them ideal for detecting subtle physiological movements such as body movement monitoring and artery pulse sensing.

Piezoelectric sensors have attracted significant attention due to their ability to generate electrical signals in response to mechanical stress. Unlike capacitive, piezoresistive, optical, and bioimpedance-based devices, piezoelectric sensors do not require an external energy source for signal generation.^5–8^ This reduces energy consumption and simplifies circuit design, making them highly efficient and practical for various applications. Poly­(vinylidene fluoride) (PVDF) and its copolymers have emerged as prominent organic piezoelectric materials in flexible piezoelectric sensors because of their flexibility, biocompatibility, and comparable piezoelectric coefficients (*d*_33_ ≈ 24–140 pC/N).^9,10^ Different strategies have been developed to enhance the piezoelectric coefficients of PVDF-based materials. Electrospinning is considered a cost-effective and easy processing method to produce large-scale self-poling PVDF-based nanofibers (NFs) with a high β-phase ratio by simultaneously applying high electrical fields and stretching forces.^11–13^ Additionally, incorporating nanofillers into PVDF to form nanocomposite materials can further improve and stabilize the β-phase conformation of PVDF through surface interactions to enhance output performance.^14–16^ As shown in Table 1, flexible electrospun composite PVDF NFs can combine the piezoelectric properties of PVDF with inorganic materials (BaTiO_3_, KNN, ZnO, etc.) to enhance piezoelectric performance for monitoring body movements. Subtle physiological signals, such as heart rate and arterial pulse, are key health indicators which require the development of high-sensitivity portable sensors that conform to the human body and are capable of monitoring low-intensity signals. Electrospun PVDF fibers, sandwiched between two nonpiezoelectric electrospun fiber mats (polyurethane and poly­(vinyl alcohol)) with electrode deposition, have been reported as effective mechanoacoustic sensors for heart monitoring due to their combined triboelectric and piezoelectric properties.^17^

**Table 1:** Comparison of the Present Work With Recent Literature Reports of Electrospun PVDF Fiber-Based Piezoelectric Sensors^24–30^

refs	year	device made up	thickness	peak output performance	sensitivity	applied forces range
^24^	2019	BTO/PVDF–TrFE	300 μm	25.6 nA; pressure ∼7 kPa		pressure range 0.77 ∼ 7.0 kPa at 10 Hz
^25^	2020	0.5Ba(Zr_0.2_Ti_0.8_)O_3_–0.5(Ba_0.7_Ca_0.3_)TiO_3_ PVDF–TrFE	40 μm	∼6 V; compressed force of 6 N at 10 Hz	150 mV/kPa	compression range 1–10 N (10–100 kPa) at 10 Hz
^26^	2020	ZnO nanorods on PVDF	35 μm	∼1.5 V; pressure of 451 kPa	3.12 mV/kPa	pressure range 1.8–451 kPa
^27^	2021	PMMA@BTO/PVDF–TrFE	20 μm	∼15 V; bended under 3 Hz, 4 mm displacement	∼4.29 V/Hz	bending range 0.25–3 Hz, 4 mm displacement
^28^	2023	PVDF–TrFE NFs sandwiched spray BTO		13.56 V; pressure at 300 kPa and 4 Hz	35.78 mV/kPa	pressure range 40–300 kPa at 4 Hz frequency
^29^	2023	Sb nanosheets/BTO nanoparticles/PVDF–TrFE NFs		∼17.1 V pressure at 128 kPa and 2 Hz	96 mV/kPa	pressure range 16–128 kPa at 2 Hz
^30^	2024	layer-by-layer Mxene/PVDF–TrFE fiber mat and boron nitride/PVDF–TrFE fiber mat		∼1 V; pressure at 50 kPa	39.3 mV/kPa (1–10 kPa), 14.94 mV/kPa (10–50 kPa)	pressure range of 1–50 kPa
this work	2025	PVDF–TrFE/3 wt % BTO	43 μm	9.8 V; pressure of 16 kPa (10 N) at 7 Hz	370 mV/kPa	pressure range 6.4–16 kPa at 7 Hz

We have shown in our previous work that triboelectric energy harvesters based on electrospun tetragonal BaTiO_3_ (BTO)/PVDF–TrFE exhibited significantly improved performance compared to earlier PVDF/BTO triboelectric energy harvesters.^18^ However, the design of triboelectric sensors, which relies on contact-separation or lateral-sliding between the surfaces of two counterpart materials, faces challenges such as effective interaction, durability, and integration issues.^19^ Therefore, a PVDF–TrFE film-based sensor with PEDOT/PSS electrodes was fabricated for pulse monitoring. The PVDF–TrFE film was prepared using a bar coating process, where the polymer solution was spread onto a substrate with a coating rod. Nonetheless, the PVDF–TrFE film still requires polarization by a high electric field to activate its piezoelectric properties.^20^ To achieve portable human movement monitoring, eliminating cumbersome wired connections is essential for improving user comfort and mobility. Hence, integrating wireless data transfer systems with flexible sensors is expected to enhance timely health monitoring and sports performance management, ensuring seamless communication within IoT networks and with remote data analysis platforms and software-based operation.

Herein, we developed a wearable and highly sensitive PVDF–TrFE (3 wt % BTO) piezoelectric sensor capable of monitoring low-frequency and pressure/force signals with significantly higher sensitivity compared to previous work. The fabricated sensor based on electrospun NFs of PVDF–TrFE with 3 wt % BTO exhibited a sensitivity of 0.37 V/kPa within a pressure range of 6.4–16 kPa at a fixed frequency of 7 Hz. These fabricated PVDF–TrFE (3 wt % BTO) piezoelectric sensors are compact with integrated functionalities and structures to make them versatile for use as wearable smart patches attached to the wrist for monitoring body movement and pulse rate. Additionally, pressure sensor arrays were fabricated using these materials, integrated with wireless data transfer systems, allowing data from specific pressure locations to be transmitted to a smartphone for real-time spatial detection of impact events. The proposed wearable PVDF–TrFE (3 wt % BTO) piezoelectric sensor and wireless data transfer system hold great potential for autonomous real-time deformation and health monitoring, crucial for assessing overall health and detecting early signs of medical disorders.

## 2 Results and Discussion

### 2.1 Morphological, Characterization and Performance of Electrospun PVDF–TrFE–BTO Fibers

Figure 1 illustrates the fabrication process of a flexible pressure sensor by electrospinning and its application to pressure sensors array and real-time body movements monitoring system. (i) The prepared solutions were electrospun by applying onto the collector through a syringe (10 mL) with a flow rate of 0.5 mL/h and a high voltage (17 kV) applied between the needle and drum collector (15 cm distance) at room temperature and humidity (see Section 4 for detailed fabrication). (ii) The electrospun NFs mat was carefully peeled off and then deposited with top and bottom electrodes to develop flexible pressure sensors. (iii) The completed piezoelectric pressure sensor was conformally attached to a human body using a widely used polyurethane waterproof bandage to monitor the biosignals, including artery pulse signal and joint movement, following wireless transmission of the detected signals to a smartphone. (iv) Pressure sensor arrays were fabricated from the above materials, enabling real-time detection of pressure from specific locations.

**Figure 1:**
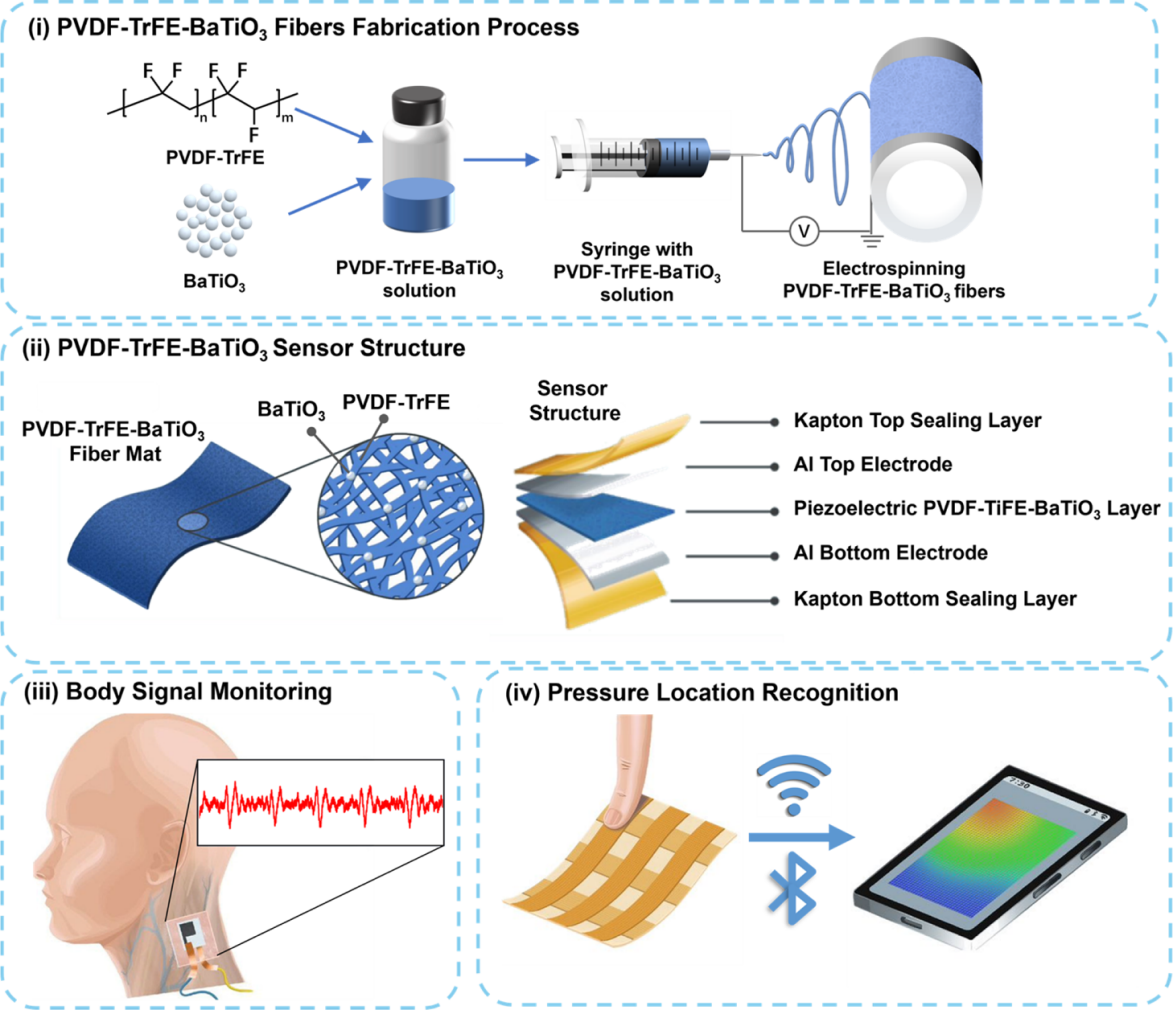
Schematic illustration of the fabrication process for nanofiber flexible sensors: (i) PVDF–TrFE–based nanofiber mats obtained by electrospinning. (ii) Flexible sensors fabricated by sandwiching PVDF–TrFE NFs between top and bottom aluminum (Al) electrodes. (iii) Sensor attached to the human body for monitoring body movement. (iv) Pressure sensor arrays fabricated, enabling wireless transmission of detected pressure signals to a smartphone.

The surface morphology of the electrospun NFs was examined using scanning electron microscopy (SEM) with a secondary electron detector. Figure 2a–d displays the SEM micrographs of the films before (Figure 2a) and after the incorporation of BTO nanoparticles into the solution (Figure 2b–d). The BTO nanoparticles are embedded within the nanofiber networks. They are visible on the fiber surfaces, becoming more prominent with increasing BTO concentration, as highlighted in the red square in Figure 2d of PVDF–TrFE (3 wt % BTO). Figure S1 presents a high-definition backscattered electron (HDBSD) SEM image of PVDF–TrFE (3 wt % BTO), revealing the distribution of BTO nanoparticles. The bright spots correspond to BTO particles, while the darker continuous strands represent the PVDF–TrFE NFs forming a nonwoven network. The dispersion of BTO on and within the fibers indicates successful composite formation during electrospinning. Figure S2a–d shows low-magnification SEM images demonstrating that four types of nanofiber mats exhibit a completely fibrous structure with uniform nanofiber distribution. Figure S2e–h shows the corresponding fibers diameter distribution with 0.24 μm, 0.24 μm, 0.26, and 0.27 μm for pure PVDF–TrFE, PVDF–TrFE with 1 wt % BTO, 2 wt % BTO, and 3 wt % BTO, named PVDF–TrFE (1 wt % BTO), PVDF–TrFE (2 wt % BTO), and PVDF–TrFE (3 wt % BTO), respectively. This indicates that the uniform distribution of NFs is maintained after the addition of BTO nanoparticles.

**Figure 2:**
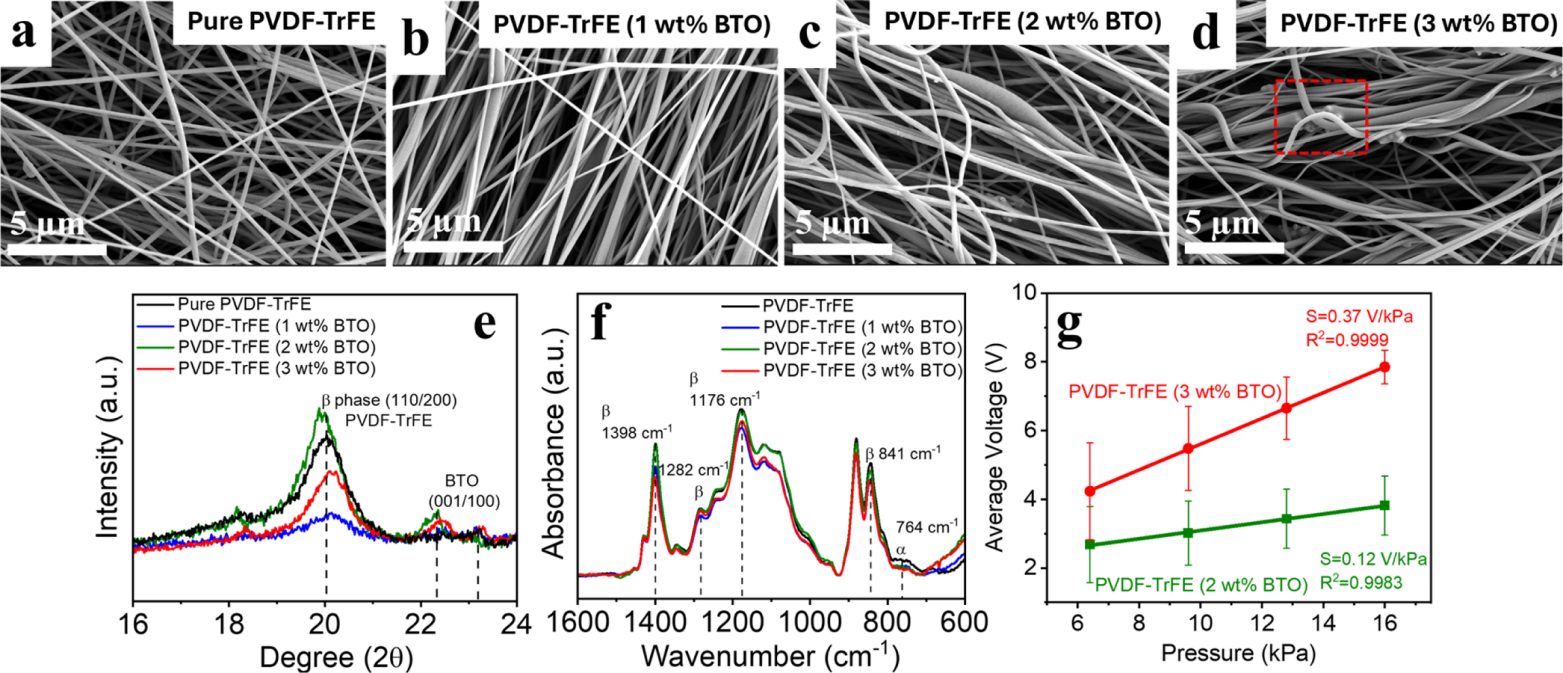
SEM images of (a) PVDF–TrFE, (b) PVDF–TrFE (1 wt % BTO), (c) PVDF–TrFE (2 wt % BTO), and (d) PVDF–TrFE (3 wt % BTO). (e) X-ray diffraction (XRD) results of PVDF–TrFE and PVDF–TrFE/BTO samples with crystalline β-phase (110/200) and XRD patterns of the tetragonal BTO nanoparticles (001/100); (f) FTIR spectra of PVDF–TrFE and PVDF–TrFE/BTO samples. (g) Pressure sensitivity of PVDF–TrFE (2 wt % BTO) sensor and PVDF–TrFE (3 wt % BTO) sensor at the constant frequency of 7 Hz and various pressures.

Powder X-ray diffraction (PXRD) patterns were used to characterize the crystallinity and β phase in the PVDF–TrFE matrix for each of the four varieties of electrospun NFs. The XRD data in Figure 2e show a dominant peak around 2θ = 20° for all four samples, corresponding to the reflection of the crystalline β-phase (110/200) of PVDF–TrFE. With increasing amounts of BaTiO_3_, the PVDF–TrFE/BTO NFs diffraction pattern (blue line) exhibits more pronounced peak splitting at 22.2°, corresponding to the (*hkl*) Miller indices (001) and (100).^21^ The full width at half-maximum (fwhm) was calculated to determine the β-phase crystalline quality of the different samples, where smaller fwhm values indicate higher β-phase crystallinity. The fwhm values for pristine PVDF–TrFE, PVDF–TrFE (1 wt % BTO), PVDF–TrFE (2 wt % BTO), and PVDF–TrFE (3 wt % BTO) are 1.52, 1.19, 1.15, and 1.06, respectively.^22^ Fourier transform infrared (FTIR) spectroscopy was also used to determine the amount of β-phase in the four types of electrospun nanofiber mats. As shown in Figure 2f, there is no obvious α-phase characteristic peak at 764 cm^–1^ due to the inherently high β-phase content of PVDF–TrFE (75/25). The content of polar phases (F­(β)) can be calculated using eq 2 shown in the Section 4.^23^ It was found that the amount of β-phase was higher for PVDF–TrFE (3 wt % BTO) (91.79%) compared to pure PVDF–TrFE (84.82%), PVDF–TrFE (1 wt % BTO) (89.70%), and PVDF–TrFE (2 wt % BTO) (90.13%). Thus, the presence of a higher crystallinity and proportion of the β-phase enhances dipole alignment and polarization in the PVDF–TrFE–BTO matrix, leading to an increase in the performance of the piezoelectric sensor. To further compare pressure sensitivity, aluminum (Al) foil was used as electrodes sandwiching the materials. Sensor performance was studied at different pressures, but at a fixed frequency of 7 Hz, using an electrodynamic shaker system. Three sets of samples: PVDF–TrFE (2 wt % BTO) and PVDF–TrFE (3 wt % BTO), were fabricated to investigate the effect of BTO concentration on device performance. Figure S3a displays the typical output voltage of the PVDF–TrFE (2 wt % BTO) sensor under cyclic compressive forces of 4, 6, 8, and 10 N, corresponding to pressures of 6.4, 9.6, 12.8, and 16 kPa, respectively. Table S1 presents the relative output parameters from three samples of PVDF–TrFE (2 wt % BTO) during testing. The output voltage increases with pressure, with a maximum average output voltage of 3.9 V at 6.4 kPa, reaching up to 4.8 V at 16 kPa. The deformation of the piezoelectric film creates a potential difference, resulting in an output voltage under increasing pressure. As shown in Figure S3b, the PVDF–TrFE (3 wt % BTO) sensor exhibited higher output voltage at the same applied pressure compared to the PVDF–TrFE (2 wt % BTO) sensor, with maximum and average output voltages of 9.8 and 8.4 V at 16 kPa, respectively. Table S2 shows that the maximum average output voltage increases from 5.7 to 8.4 V as pressure increases from 6.4 to 16 kPa. The inset picture of Figure S3 also demonstrates the high flexibility of the fabricated pressure sensor, as it can be rolled around a pen.

Figure 2g illustrates the sensitivity of the sensor based on PVDF–TrFE with 2 and 3 wt % BTO nanofiber mats. The results indicate a linear increase in output voltage with applied pressure. The average output voltage of PVDF–TrFE (2 wt % BTO) and PVDF–TrFE (3 wt % BTO) increases from 2.68 to 3.82 V and 4.23 to 7.85 V, respectively, as pressure increases from 6.4 to 16 kPa. This suggests that the enhanced crystallinity and higher proportions of β-phase in PVDF–TrFE (3 wt % BTO) contribute to increasing the sensor’s performance. The calculated pressure sensitivity for the PVDF–TrFE (3 wt % BTO) sensor is 0.37 V/kPa, higher than the 0.12 V/kPa sensitivity of the PVDF–TrFE (2 wt % BTO) sensor. Table 1 compares the output performance of electrospun PVDF fiber-based piezoelectric sensors reported in the literature to date.^24–30^ It was found that the PVDF–TrFE (3 wt % BTO) sensor performs well under moderate pressure and frequencies compared to the literature, demonstrating its ability to detect small-regime mechanical forces and its potential applications.

### 2.2 Application of the PVDF–TrFE (3 wt % BTO) Sensor for Body Signals Monitoring

Previous studies have shown that signals generated by human body are mainly distributed across low-pressure (1–10 kPa) and medium pressure (10–100 kPa) ranges.^31^ For instance, heart signals typically fall within a frequency range of 10–250 Hz and pressures below 10 kPa;^17,32^ relaxed finger movements occur at frequencies below 10 Hz and pressures under 100 kPa;^33^ and body movements are characterized by frequencies below 5 Hz and pressures ranging from 100 to 370 kPa.^34^ These signals are generally associated with low frequency and low intensity.^35^ Herein, PVDF-–TrFE (3 wt % BTO) sensor was further tested under low pressure and frequency at fixed frequency and pressure. Figure 3a shows the output voltage generated by PVDF–TrFE (3 wt % BTO) at increasing pressures from 6.4 to 22.4 kPa at a fixed frequency of 2 Hz. Table S3 summarizes the output voltage parameters recorded under different pressures. The PVDF–TrFE (3 wt % BTO) sensor can produce the maximum output voltage of 3.2 V at a minimum pressure of 4 N (6.4 kPa) at the low frequency of 2 Hz. With increasing applied pressure, the maximum output voltage can reach up to 4.0, 4.6, 4.8, 5.8, and 6.4 V at 6 N (9.6 kPa), 8 N (12.8 kPa), 10 N (16 kPa), 12 N (19.2 kPa), and 14 N (22.4 kPa), respectively. Figure 3b shows a linear relationship between voltage and applied pressure at a fixed frequency of 2 Hz, indicating that the output performance is roughly linear, with a sensitivity of 0.18 V/kPa. In addition, the frequency response of PVDF–TrFE (3 wt % BTO) at a fixed pressure was tested, as shown in Figure 3c,d. Table S4 summarizes the output voltage parameters at different frequencies under a fixed pressure of 4 N (6.4 kPa). As shown in Figure 3c, the output voltage of the PVDF–TrFE (3 wt % BTO) sensor is influenced by frequency at a constant pressure of 4 N (6.4 kPa). The maximum voltage increased from 4.2 to 6.4 V as the frequency increased from 4 to 7 Hz. This variation influences the piezoelectric response of the PVDF–TrFE (3 wt % BTO) sensor at applied pressure and frequency. The device exhibits linear output response characteristics under both low pressure and frequency regimes, with enhanced piezoelectric response at higher frequencies, improving its sensitivity to external vibrations. This demonstrates that our piezoelectric PVDF–TrFE (3 wt % BTO) flexible sensor can identify low-regime biosignals arising from biomechanical movements by converting them into electric energy.

**Figure 3:**
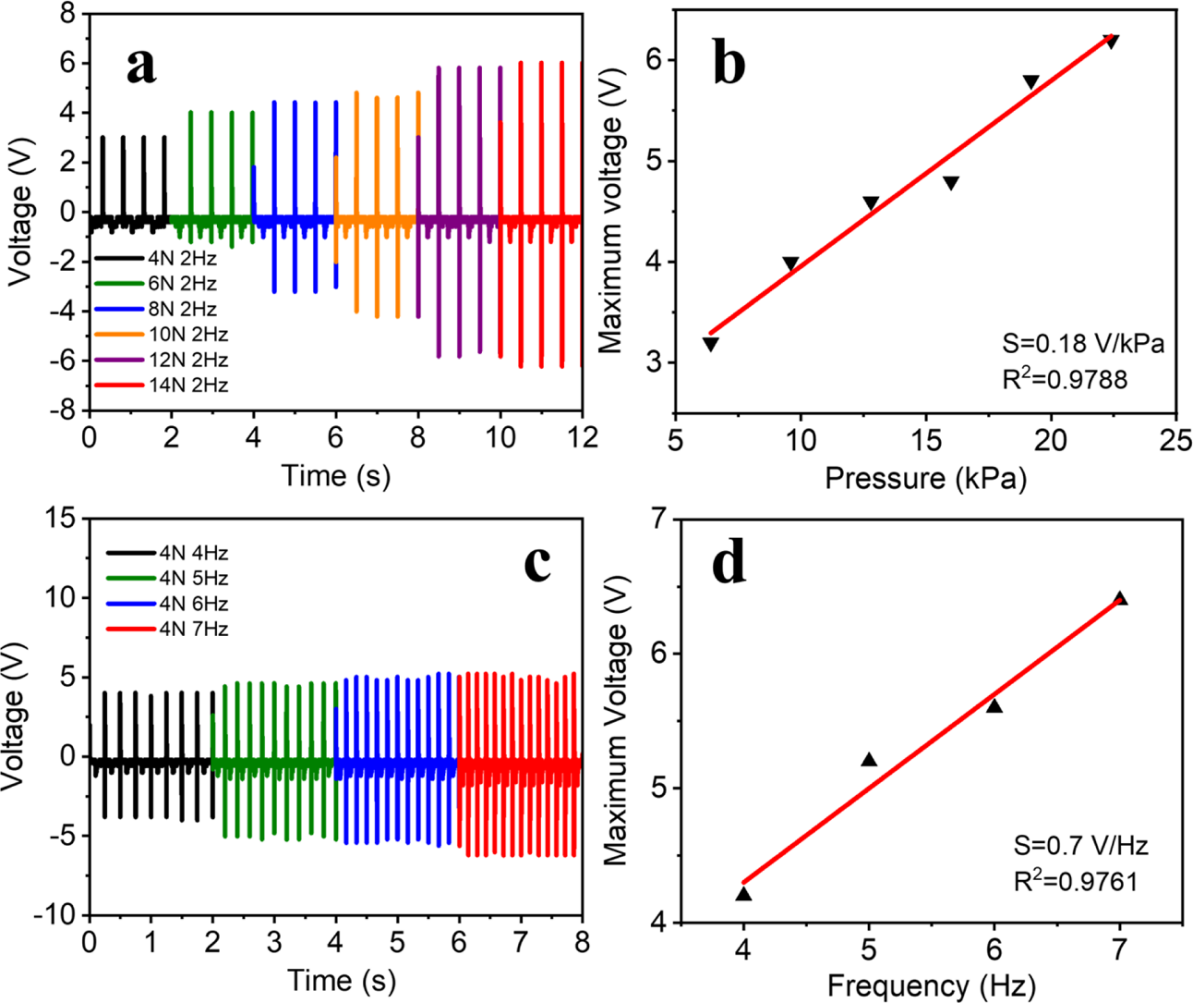
(a) Typical output voltage signal and (b) pressure sensitivity of PVDF–TrFE (3 wt % BTO) under different pressures at a constant frequency of 2 Hz. (c) Typical output voltage signal and (d) frequency sensitivity of PVDF–TrFE (3 wt % BTO) under different frequencies at a constant pressure of 4 N (6.4 kPa).

To detect subtle physiological movements, the sensor must be attached conformally to the complex contours of the human body. Herein, Al electrodes were deposited on the top and bottom sides of electrospun PVDF–TrFE (3 wt % BTO) fibers using a thermal evaporator. The electrode area was 1.5 cm × 2.5 cm, as shown in Figure 4a. SEM and the elemental mapping were exploited to study the microstructures and morphologies of the edge between the Al-coated and uncoated surfaces (Figure 4b,c). The corresponding elemental mapping of Al and carbon (C) further verifies that Al is coated on the surface of electrospun PVDF–TrFE (3 wt % BTO) fibers. Figure 4d shows layered elemental mapping depicting the elemental distribution of an enlarged Al-coated surface. The elemental mappings of C, F, and Al (Figure 4e–g) reveal colocalized patterns corresponding to the electrospun fibers, including surface deposition of Al. Furthermore, Figure 4h,i indicate the presence of Ba and Ti elements in similar areas as nanoparticles on the surface of the electrospun NFs, consistent with SEM observations. Figure S4 shows the performance of electrospun PVDF–TrFE fibers with thermally deposited Al electrodes versus those with Al foil electrodes of the same dimension. The devices were tested under subtle frequencies from 2 to 5 Hz at a fixed pressure of 3.2 kPa. The peak-to-peak voltage from the device with deposited Al electrodes is higher than that from the device with Al foil electrodes, indicating that devices with integrated deposited electrodes work well for mechanical stimulation detection. The fabricated sensor was then transferred to a transparent bandage film for conformal attachment to the human neck to test the carotid artery pulse, which is easily accessible and strong. The measured signals were compared to the pulse waveform signal recorded with a commercial electrocardiogram (ECG) placed on the wrist, as shown in the inset digital pictures of Figure 4j. Electrical signals generated by the heart during each beat propagate throughout the body and can be detected by ECG electrodes placed on the wrist. In our study, one electrode was placed on the left wrist, another on the right wrist, and a reference electrode on the abdomen. The experiments were performed on a healthy female subject in her early thirties with no history of cardiovascular disease or arrhythmias. No allergic reactions, skin wounds, or adverse effects were observed throughout the study. Recording electrical signals through ECG requires at least three electrode attachments, which prevents continuous monitoring and introduces discomfort and inconvenience for users. However, our fabricated sensor relies on measuring the biomechanical force generated by the arterial pressure wave. This approach eliminates the need for multiple electrode placements and is easy to fabricate, cost-effective, and conformable to the skin. Moreover, the sensor provides direct and highly sensitive measurements of physiological events, making it a practical alternative for wearable health monitoring systems. As shown in Figure 4j, the pulse data collected from the PVDF–TrFE (3 wt % BTO) sensor is within the expected and normal range, with an average peak-to-peak voltage of 30 mV and approximately 65 beats per minute (BPM) generated from the radial artery pulse. The measured values are close to those of a commercial ECG sensor, demonstrating the prepared device’s ability for real-time physiological monitoring. Thus, the PVDF–TrFE (3 wt % BTO) sensor can be used as a biomedical sensor for continuous monitoring of pulse waves and IoT-based remote healthcare clouds. In addition, the fabricated sensor can be attached to the human wrist to test wrist bending, as shown in the inset picture of Figure 4k. The current–time scans of this device during gentle wrist bending were measured on a source-measure unit (Keithley 2400), with a current ranging between 0.05 and −0.15 μA, indicating its ability to monitor body movement.

**Figure 4:**
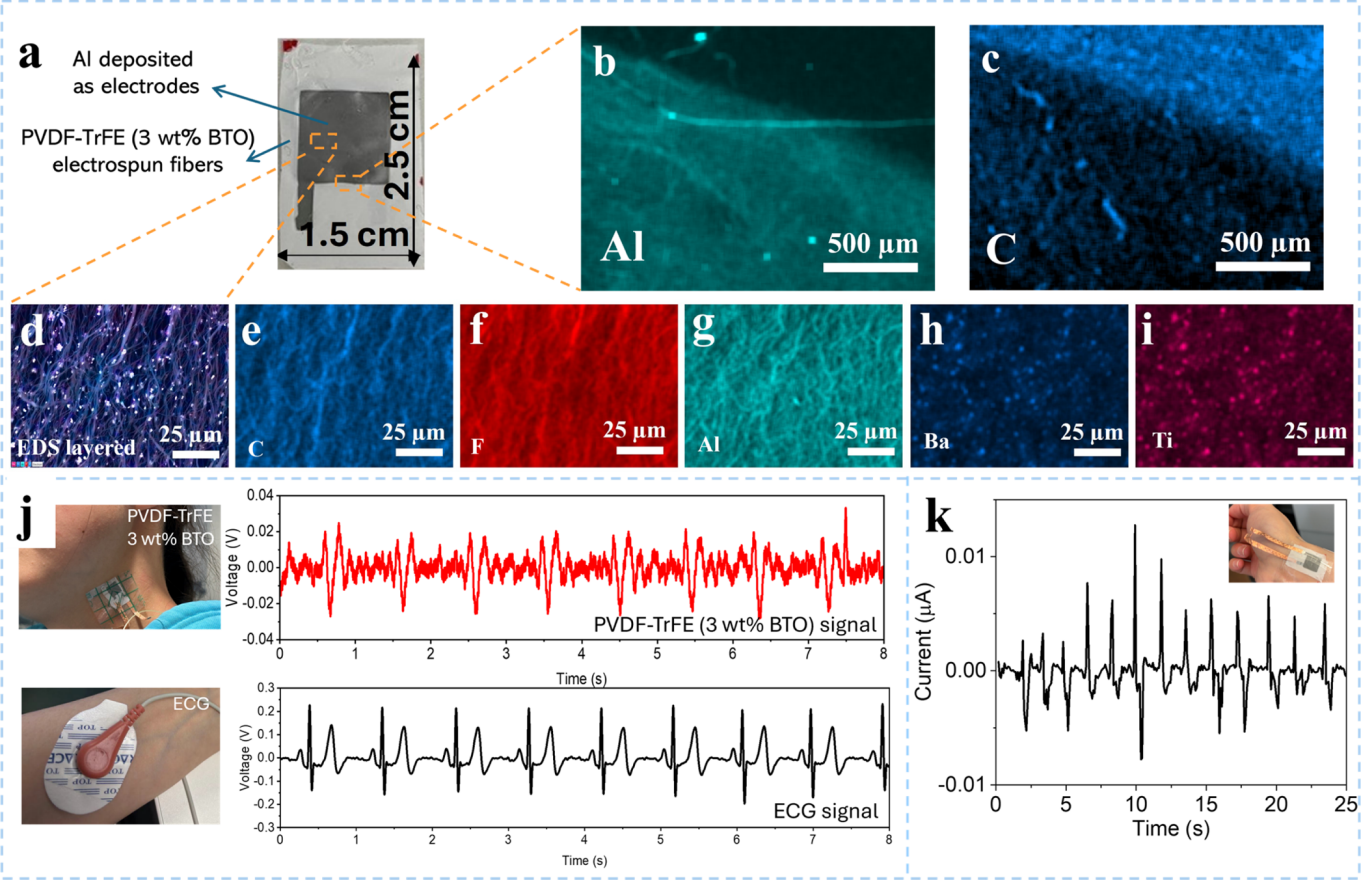
PVDF–TrFE (3 wt % BTO) sensor for biomechanical signal monitoring. (a) Optical photograph of the sensor. (b,c) SEM image and elemental distribution mapping of Al and C between Al-coated and uncoated surfaces. (d) Layered elemental mapping of the enlarged Al-coated surface and corresponding elemental distribution of (e) C; (f) F; (g) Al; (h) Ba; (i) Ti. (j) Comparison of arterial pulse signals from a commercial ECG on the wrist and the PVDF–TrFE (3 wt % BTO) sensor on the neck. (k) Current–time scan of the device under wrist bending, with an inset showing the device on the wrist.

### 2.3 Wireless Data System and Sensor Arrays for Pressure Recognition

The wireless data system was designed to transfer the signal from our fabricated flexible PVDF–TrFE (3 wt % BTO) sensor to a portable device for IoT applications. Figure 5a shows the wireless data transfer scheme. In practice, the flexible PVDF–TrFE (3 wt % BTO) sensor generates a voltage signal, converted to a digital signal and transmitted to personal devices (e.g., phones, tablets, smartwatches, fitness trackers) via a Bluetooth module. Figure 5b displays the optical photographs of a portable signal detection and data transfer system, including a portable battery of 3.7 V and 500 mAh, nRF52840 Bluetooth module and a mobile phone. Here, the signal from the PVDF–TrFE (3 wt % BTO) sensor recorded by tapping is displayed and collected by software. The piezoelectric signals for manual ‘finger pressing’ are rectified and collected through an app on a mobile phone, as illustrated in Figure 5c. Where the positive output voltage is similar to oscilloscope measurements (Figure S5). It is worth noting that pressure sensor arrays are ideal for monitoring external impacts and recognizing pressure positions, meeting wearable electronics requirements for body movement monitoring.

**Figure 5:**
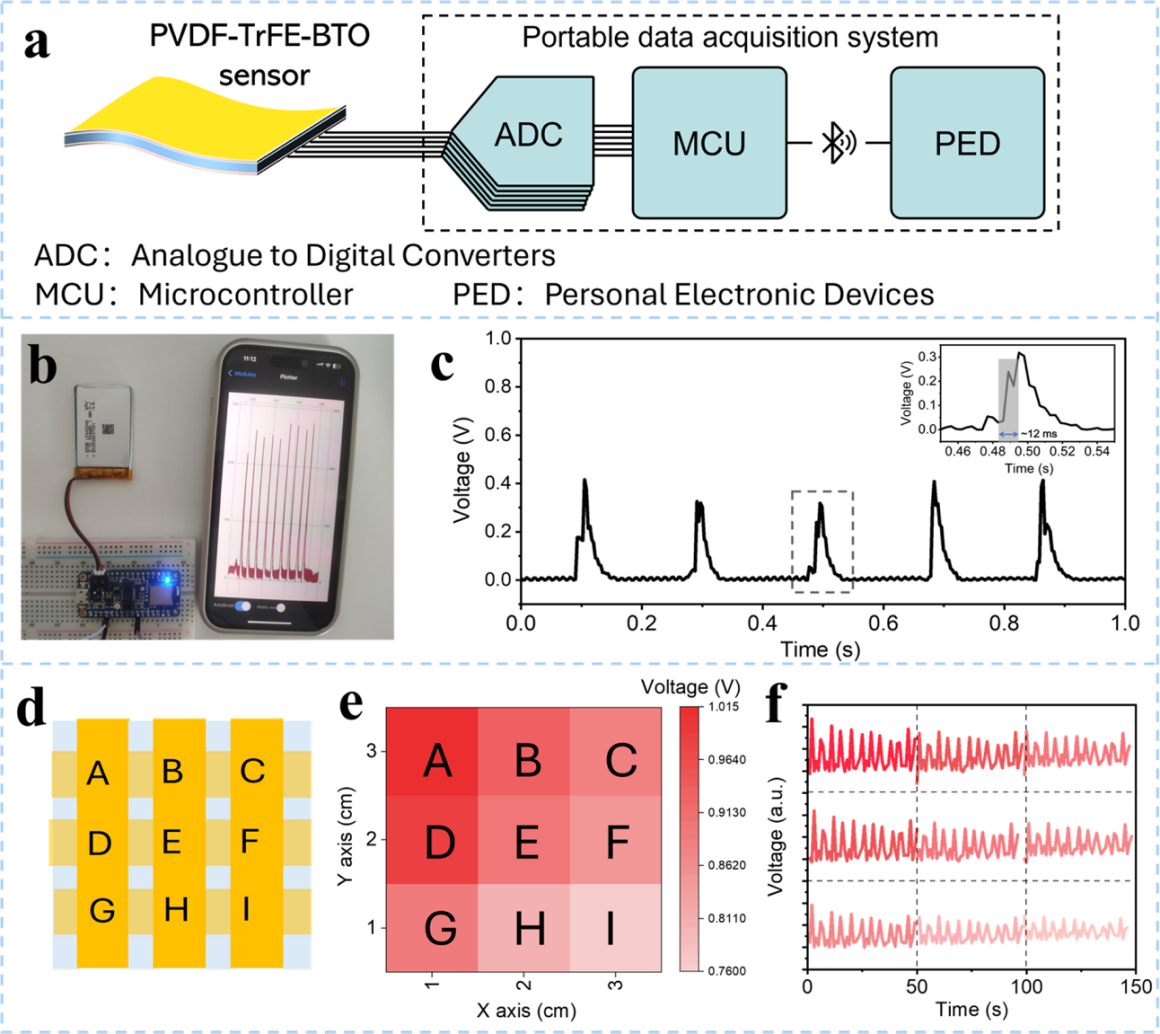
PVDF–TrFE–BTO_3_ sensor application and wireless data transfer for IoT. (a) Illustration of the PVDF–TrFE (3 wt % BTO) sensor and wireless data transfer system. (b) Photograph of the portable data acquisition system. (c) Press signal from tactile sensing wirelessly transferred to a mobile phone via a microcontroller. (d) Schematic of the pressure sensor array based on electrospun PVDF–TrFE (3 wt % BTO). (e) Contour map of average voltage amplitudes. (f) Real-time arterial voltage signals measured at each pixel under tactile tapping.

Pressure sensor arrays based on PVDF–TrFE (3 wt % BTO) were fabricated, as shown in Figure 5d. The 3 × 3 active-matrix sensor arrays divide the panel into 9 positions (A to I) as a proof-of-concept for a “pressing touching board”. Figure 5e displays contour maps of voltage output signals from finger touching at position A, highlighting voltage amplitude differences by position. The position that exhibits higher voltage outputs compared to other pixels indicates the position where pressure is applied. Figure 5f shows the real-time average output voltage amplitudes measured at each position during finger pressing at position A. The outputs result from vibration propagation through the whole panel during pressing. These data suggest that the output is proportional to the distance from the pressing point, with the highest voltage amplitude for position A, indicating higher pressure at A, gradually decreasing with distance. Likewise, positions B and D, closest to A, show higher voltage amplitudes, while positions further away exhibit gradually reducing signals. Based on these measurements, logic codes (“0”, “1”) were assigned to determine pressure location on the device. As demonstrated in Video 1, the pressing-induced voltage signal was converted to a digital signal and wirelessly transmitted to a mobile phone via a Bluetooth module, allowing for position detection and recognition.

The output levels provided are illustrative examples produced by our current device. Future devices will need calibration to determine tolerance levels for each output code. Video 1 showing position detection and recognition provides clear evidence that the results achieved in this work demonstrate the potential to produce a fully integrated impact sensing system with intrinsic sensing ability and spatial recognition capability. Our results demonstrate a sensor positioning system utilizing an array of pressure sensors, featuring fully integrated wireless data transfer and collection, which enables intrinsic sensing and spatial recognition. As such, the method proposed enhances the sensitivity and functionality of electrospun PVDF–TrFE–BTO sensors. Here, this proof-of-concept demonstrates the potential to produce a wearable monitoring system capable of transmitting real-time sensing data to portable devices. While we have demonstrated the concept here with just prototyping device, the concept could be extended to multiple array matrices, device miniaturization and ergonomic optimization. This would enable accurate monitoring of heartbeats, pressure variations, and spatial distribution of forces during user movement or running while mitigating the influence of environmental factors (e.g., humidity, sweat, and temperature), making it suitable for wearable applications in IoT and smart surfaces in a variety of touch sensor devices.

## 3 Conclusion

This work reports a flexible PVDF–TrFE–BTO nanofiber-based piezoelectric sensor. PVDF–TrFE–BTO NFs were successfully fabricated by electrospinning, and the effect of BTO concentration on the crystallinity and β phase proportion was investigated. The piezoelectric sensor based on PVDF–TrFE (3 wt % BTO) NFs exhibited good pressure and frequency sensitivity at low mechanical forces due to higher crystallinity and β phase proportion, with a pressure sensitivity of 0.37 V/kPa under 16 kPa and 7 Hz.

The sensor is characterized by a good linear response to varying pressures and frequencies, showing that the performance achieved is comparable to that reported in recent literature. A wireless data transfer system was developed to convert detected voltage signals to digital signals for transmission to portable electronic devices. It was demonstrated that the PVDF–TrFE (3 wt % BTO) sensor developed can be utilized to detect physical motions and physiological signals, with wearable patches on the wrist and neck for bending detection and pulse monitoring. Furthermore, we have established that the sensor functions as a pressure-sensing board, capable of detecting external pressure forces and locations, thereby demonstrating an addressable sensor array. The PVDF–TrFE (3 wt % BTO) sensor, designed and fabricated, was characterized by high-sensitivity detection for biomechanical signals. The integrated wireless data transmission system designed in this work, holds significant potential for applications in portable electronics, particularly for real-time health monitoring.

## 4 Experimental Section

### 4.1 Preparation of Materials

A 15 wt % (PVDF–TrFE) solution was prepared by dissolving PVDF–TrFE powder (75/25, Piezotech, France) in a 1:1 DMF and acetone (Sigma-Aldrich, UK) solvent mixture, stirred for 10 h at 300 rpm and 25 °C. The solution was stirred until it became homogeneous and completely transparent. PVDF–TrFE and BaTiO_3_ (BTO) (Tetragonal, Sigma-Aldrich, UK) composite solutions were then prepared by mixing the (PVDF–TrFE) solution with 1, 2, and 3 wt % BTO, respectively. These nanocomposite solutions were stirred at 300 rpm and 25 °C for 4 h to uniformly disperse the BTO, forming PVDF–TrFE/BTO slurries with different BTO concentrations.

### 4.2 Electrospinning

Electrospinning of the NFs was performed using an electrospinning machine (TL-Pro, Tongli Tech.). The prepared solutions were loaded into a 10 mL syringe connected to a spinneret with a 21 G-gauge needle. Electrospinning was subsequently carried out at 17 kV and a flow rate of 0.5 mL/h, with a 15 cm distance between the needle and drum collector. The electrospun NFs were collected on a drum collector covered with aluminum foil (Sigma-Aldrich, UK), rotating at 1500 rpm and 2 kV voltage. All four polymer solutions (PVDF–TrFE, PVDF–TrFE (1 wt % BTO), PVDF–TrFE (2 wt % BTO), and PVDF–TrFE (3 wt % BTO)) were electrospun under the same conditions at room temperature (20.5 °C) and 36% humidity.

### 4.3 Device Fabrication and Performance Tests

The electrospun NF mats on the Al foil were cut into small pieces for various measurements. To measure the piezoelectric response of PVDF–TrFE–BTO sensors with different BTO concentrations, the NF mat was sandwiched between two Al foils (4.8 × 2.5 cm) and encapsulated with polyimide tape. To fabricate wearable sensors, Al electrodes with a thickness of ∼150 nm were deposited on both sides of the NFs mat surface using a vacuum thermal evaporator (E306A, Edwards) (1.5 × 1.5 cm). A shadow mask was used to prevent Al deposition near the substrate edges to avoid short circuits. Flat copper ribbons were bonded to the NFs and Al contacts using silver paste adhesive. Three samples were fabricated for each parameter to evaluate and compare performance. The fabricated sensors were tested under various pressures and frequencies using an electrodynamic shaker system (TIRA, TV 50018, Germany) powered by a power amplifier. This system generates programmable mechanical vibrations through electromagnetic excitation, accurately simulating dynamic loading environments. The output voltage was recorded using a digital storage oscilloscope (DSOX3014T). To test the biosignal from human body movement, the flexible sensor with an Al electrode (without polyimide encapsulation) was transferred onto a transparent bandage film to ensure a conformal attachment to the body, thereby enabling the recording of pulse signals and wrist movements.

### 4.4 Characterization

To investigate the surface morphology and compositional contrast of the films produced, field emission scanning electron microscopy (FESEM) micrographs of the NFs were obtained using a FEI Helios Nanolab 600, operated in secondary electron (SE2) mode at 5 kV and high-definition backscattered electron (HDBSD) mode at 20 kV. Powder X-ray diffraction (PXRD) patterns were recorded on a Bruker D8 Advance in Bragg–Brentano geometry with a Lynx-eye detector and Cu Kα radiation. The average crystallite size was estimated according to Scherrer’s equation given by eq 1,^36^

1 $$D_{hkl}=\frac{K\lambda }{\beta_{hkl}\cos\theta }$$

where *D*_*hkl*_ is the average crystallite size along the (110)/(200)_β_ crystal plane of PVDF–TrFE, *K* is the shape factor (which varies with the crystallite shape. 0.89 was chosen here), λ is the wavelength of the incident X-rays, β_*hkl*_ is the full width at half-maximum of the (110)/(200)_β_ reflection, θ is the diffraction angle.

A PerkinElmer frontier Fourier transform infrared spectrometer (FTIR) system equipped with a Quest Specac attachment was used to determine chain conformations and crystalline phases. The relative fraction of β phase content in films (containing both α and β phases) was calculated using eq 2, derived from the Lambert–Beer Law, where *A*_α_ and *A*_β_ are the absolute intensities of the peaks at 764 cm^–1^ and 841 cm^–1^, respectively.^23^

2 $$F\left(\beta \right)=\frac{A_{\beta }}{1.26A_{\alpha }+A_{\beta }}$$

### 4.5 Body Signal Monitoring Measurement

#### 4.5.1 Wrist Joint Movement Monitoring

The output current signals by wrist joint bending were acquired via a programmable source-measure unit (Keithley 2400 SMU). The software platform is constructed based on NI LabVIEW and is capable of performing real-time data collection and analysis.

#### 4.5.2 Arterial Pulse Wave Measurements

Arterial pulse wave measurements were carried out by attaching the fabricated sensors to the carotid pulse on the neck, just below the jawline. An ultrathin transparent bandage tape was gently applied to secure the sensor without exerting pressure on the artery. For reference, three electrodes of a commercial electrocardiogram (ECG) were also attached to the subject’s right wrist, left wrist, and abdomen simultaneously. Data were recorded at a sampling rate of 10 kHz using a digital data acquisition (PowerLab C, ADinstrument). The subject was comfortably seated on a chair with a backrest support. A digital filter with a 2–20 Hz bandwidth was applied to the signals from both the ECG and fabricated sensors to remove noise and improve the clarity of the biosignals recorded. The study protocol was thoroughly reviewed and approved by the ethical committee of Durham University.

## Supplementary Material





## Notes

The pressing-generated voltage is wirelessly transmitted via Bluetooth to a smartphone for location detection (MP4)
High-definition backscattered electron (HDBSD) SEM image of PVDF–TrFE (3 wt % BTO); SEM images under low magnification and diameter distribution of pure electrospun PVDF–TrFE nanofiber and electrospun PVDF–TrFE with 1–3 wt % BTO nanoparticles; typical output voltage signal of PVDF–TrFE (2 wt % BTO) sensor and PVDF–TrFE (3 wt % BTO) sensor at a fixed frequency of 7 Hz and various compressive forces; output voltage parameters comparison of PVDF–TrFE (2 wt % BTO) and PVDF–TrFE (3 wt % BTO) under different pressures; output voltage parameters of PVDF–TrFE (3 wt % BTO) under different pressures but at a constant frequency of 2 Hz; output voltage parameters of PVDF–TrFE (3 wt % BTO) under different frequencies at a constant pressure of 4 N (6.4 kPa); output voltage signal from devices with deposited Al and Al foil electrode at different frequencies but a fixed pressure of 2 N (3.2 kPa); output voltage signal from finger pressing measured on an oscilloscope (PDF)

## References

[1] Jiang D., Shi B., Ouyang H., Fan Y., Wang Z. L., Li Z. (2020). Emerging implantable
energy harvesters and self-powered implantable medical electronics. ACS Nano.

[2] Jiang Q., Antwi-Afari M. F., Fadaie S., Mi H.-Y., Anwer S., Liu J. (2024). Self-powered wearable Internet of Things sensors for human-machine
interfaces: A systematic literature review and science mapping analysis. Nano Energy.

[3] Wu P., Zhao C., Cui E., Xu S., Liu T., Wang F., Lee C., Mu X. (2024). Advances in
magnetic-assisted
triboelectric nanogenerators: structures, materials and self-sensing
systems. Int. J. Extreme Manuf..

[4] Yang Y., He T., Ravindran P., Wen F., Krishnamurthy P., Wang L., Zhang Z., Kumar P. P., Chae E., Lee C. (2024). All-organic transparent
plant e-skin for noninvasive phenotyping. Sci.
Adv..

[5] Hosseini E. S., Dervin S., Ganguly P., Dahiya R. (2021). Biodegradable Materials
for Sustainable Health Monitoring Devices. ACS
Appl. Bio Mater..

[6] Qin J., Yin L.-J., Hao Y.-N., Zhong S.-L., Zhang D.-L., Bi K., Zhang Y.-X., Zhao Y., Dang Z.-M. (2021). Flexible and stretchable
capacitive sensors with different microstructures. Adv. Mater..

[7] Chowdhury A. H., Jafarizadeh B., Baboukani A. R., Pala N., Wang C. (2023). Monitoring
and analysis of cardiovascular pulse waveforms using flexible capacitive
and piezoresistive pressure sensors and machine learning perspective. Biosens. Bioelectron..

[8] He Q., Li X., Zhang H., Briscoe J. (2023). Nano-Engineered Carbon Fibre-Based
Piezoelectric Smart Composites for Energy Harvesting and Self-Powered
Sensing. Adv. Funct. Mater..

[9] Mahapatra S. D., Mohapatra P. C., Aria A. I., Christie G., Mishra Y. K., Hofmann S., Thakur V. K. (2021). Piezoelectric Materials for Energy
Harvesting and Sensing Applications: Roadmap for Future Smart Materials. Adv. Sci..

[10] Mokhtari F., Samadi A., Rashed A. O., Li X., Razal J. M., Kong L., Varley R. J., Zhao S. (2025). Recent progress
in
electrospun polyvinylidene fluoride (PVDF)-based nanofibers for sustainable
energy and environmental applications. Prog.
Mater. Sci..

[11] Mi H. Y., Jing X., Zheng Q. F., Fang L. M., Huang H. X., Turng L. S., Gong S. Q. (2018). High-performance
flexible triboelectric
nanogenerator based on porous aerogels and electrospun nanofibers
for energy harvesting and sensitive self-powered sensing. Nano Energy.

[12] Fastier-Wooller J. W., Lyons N., Vu T.-H., Pizzolato C., Rybachuk M., Itoh T., Dao D. V., Maharaj J., Dau V. T. (2024). Flexible iron-on sensor embedded
in smart sock for
gait event detection. ACS Appl. Mater. Interfaces.

[13] Fastier-Wooller J. W., Vu T.-H., Nguyen H., Nguyen H.-Q., Rybachuk M., Zhu Y., Dao D. V., Dau V. T. (2022). Multimodal fibrous static and dynamic
tactile sensor. ACS Appl. Mater. Interfaces.

[14] He Q. R., Briscoe J. (2024). Piezoelectric Energy
Harvester Technologies: Synthesis,
Mechanisms, and Multifunctional Applications. ACS Appl. Mater. Interfaces.

[15] Park Y., Shin Y. E., Park J., Lee Y., Kim M. P., Kim Y. R., Na S., Ghosh S. K., Ko H. (2020). Ferroelectric
Multilayer Nanocomposites with Polarization and Stress Concentration
Structures for Enhanced Triboelectric Performances. ACS Nano.

[16] Lan B., Zhong C., Wang S., Ao Y., Liu Y., Sun Y., Yang T., Tian G., Huang L., Zhang J. (2024). A highly sensitive coaxial nanofiber mask for respiratory monitoring
assisted with machine learning. Adv. Fiber Mater..

[17] Nayeem M. O. G., Lee S., Jin H., Matsuhisa N., Jinno H., Miyamoto A., Yokota T., Someya T. (2020). All-nanofiber-based,
ultrasensitive, gas-permeable mechanoacoustic sensors for continuous
long-term heart monitoring. Proc. Natl. Acad.
Sci. U.S.A..

[18] Min G., Pullanchiyodan A., Dahiya A. S., Hosseini E. S., Xu Y., Mulvihill D. M., Dahiya R. (2021). Ferroelectric-assisted high-performance
triboelectric nanogenerators based on electrospun P­(VDF-TrFE) composite
nanofibers with barium titanate nanofillers. Nano Energy.

[19] Wang Z. L. (2014). Triboelectric
nanogenerators as new energy technology and self-powered sensors -
Principles, problems and perspectives. Faraday
Discuss..

[20] Laurila M.-M., Peltokangas M., Montero K. L., Verho J., Haapala M., Oksala N., Vehkaoja A., Mäntysalo M. (2022). Self-powered,
high sensitivity printed e-tattoo sensor for unobtrusive arterial
pulse wave monitoring. Nano Energy.

[21] Yoon D.-H. (2006). Tetragonality
of barium titanate powder for a ceramic capacitor application. J. Ceram. Process. Res..

[22] Indolia A. P., Gaur M. S. (2013). Investigation of structural and thermal characteristics
of PVDF/ZnO nanocomposites. J. Therm. Anal.
Calorim..

[23] Gregorio R., Cestari M. (1994). Effect of Crystallization
Temperature on the Crystalline
Phase Content and Morphology of Poly­(Vinylidene Fluoride). J. Polym. Sci., Part B: Polym. Phys..

[24] Hu X. H., Yan X., Gong L. L., Wang F. F., Xu Y. H., Feng L., Zhang D. Y., Jiang Y. G. (2019). Improved Piezoelectric Sensing Performance
of P­(VDF-TrFE) Nanofibers by Utilizing BTO Nanoparticles and Penetrated
Electrodes. ACS Appl. Mater. Interfaces.

[25] Liu J., Yang B., Lu L., Wang X., Li X., Chen X., Liu J. (2020). Flexible and
lead-free piezoelectric
nanogenerator as self-powered sensor based on electrospinning BZT-BCT/P­(VDF-TrFE)
nanofibers. Sens. Actuators, A.

[26] Yang T., Pan H., Tian G., Zhang B., Xiong D., Gao Y., Yan C., Chu X., Chen N., Zhong S. (2020). Hierarchically
structured PVDF/ZnO core-shell nanofibers for self-powered physiological
monitoring electronics. Nano Energy.

[27] Shi K., Chai B., Zou H., Shen P., Sun B., Jiang P., Shi Z., Huang X. (2021). Interface induced performance
enhancement in flexible BaTiO_3_/PVDF-TrFE based piezoelectric
nanogenerators. Nano Energy.

[28] Mirjalali S., Bagherzadeh R., Mahdavi Varposhti A., Asadnia M., Huang S., Chang W., Peng S., Wang C.-H., Wu S. (2023). Enhanced piezoelectricity
of PVDF-TrFE nanofibers by intercalating with electrosprayed BaTiO_3_. ACS Appl. Mater. Interfaces.

[29] Wang J., Zhao C., Cao C., Liu M., Liu Z., Zhou P., Wang G., Zhang T., Zhang L., Qi Y. (2024). Boosting Sensing Performance of Flexible
Piezoelectric Pressure Sensors
by Sb Nanosheets and BaTiO_3_ Nanoparticles Co-Doping in
P­(VDF-TrFE) Nanofibers Mat. Adv. Electron. Mater..

[30] Tian G., Deng W., Yang T., Zhang J., Xu T., Xiong D., Lan B., Wang S., Sun Y., Ao Y. (2024). Hierarchical piezoelectric composites for noninvasive
continuous cardiovascular monitoring. Adv. Mater..

[31] Mannsfeld S. C. B., Tee B. C. K., Stoltenberg R. M., Chen C. V. H. H., Barman S., Muir B. V. O., Sokolov A. N., Reese C., Bao Z. N. (2010). Highly sensitive flexible pressure
sensors with microstructured
rubber dielectric layers. Nat. Mater..

[32] Sadek I., Biswas J., Abdulrazak B. (2019). Ballistocardiogram
signal processing:
a review. Health Inf. Sci. Syst..

[33] Mariello M., Fachechi L., Guido F., De Vittorio M. (2021). Conformal,
Ultra-thin Skin-Contact-Actuated Hybrid Piezo/Triboelectric Wearable
Sensor Based on AlN and Parylene-Encapsulated Elastomeric Blend. Adv. Funct. Mater..

[34] Yu J., Hou X., Cui M., Zhang S., He J., Geng W., Mu J., Chou X. (2019). Highly skin-conformal wearable tactile sensor based
on piezoelectric-enhanced triboelectric nanogenerator. Nano Energy.

[35] Trung T. Q., Lee N. E. (2016). Flexible and Stretchable
Physical Sensor Integrated
Platforms for Wearable Human-Activity Monitoring and Personal Healthcare. Adv. Mater..

[36] Ren X., Meng N., Zhang H., Wu J., Abrahams I., Yan H., Bilotti E., Reece M. J. (2020). Giant energy storage density in PVDF
with internal stress engineered polar nanostructures. Nano Energy.

