# Supplementary material for: A Wearable and Highly Sensitive PVDF–TrFE–BaTiO3 Piezoelectric Sensor for Wireless Monitoring of Arterial Signal
Source: ACS Appl Electron Mater. 2025 Jul 14;7(16):7562–71. doi: 10.1021/acsaelm.5c00548 (PMC12392445; doi:10.1021/acsaelm.5c00548)
Supplement: Supplementary file 2 [file el5c00548_si_002.pdf]

Supporting Information

A wearable and highly sensitive PVDF-TrFE-$BaTiO_3$ piezoelectric sensor for wireless monitoring of arterial signal

Qinrong He [a,b*], Huxi Wang [c], Jungang Zhang [c], Negin Ghahremani Arekhloo [c], Xenofon Karagiorgis [c,d], Bhavani Prasad Yalagala [c], Peter J. Skabara [d], Hadi Heidari [c], Dagou A. Zeze [a], Ensieh Hosseini[a*]

[a] Department of Engineering, Durham University, DH1 3LE Durham, U.K.

[b] Key Laboratory of Bionic Engineering (Ministry of Education), College of Biological and Agricultural Engineering, Jilin University, Changchun, Jilin, 130022, China

[c] James Watt School of Engineering, University of Glasgow, G12 8QQ Glasgow, U.K.

[d] School of Chemistry, University of Glasgow, G12 8QQ Glasgow, UK

**Corresponding author email: ensieh.hosseini@durham.ac.uk, qinrong.he@durham.ac.uk*

Supporting Information

High-definition backscattered electron (HDBSD) SEM image of PVDF-TrFE (3 wt% BTO); SEM images under low magnification and diameter distribution of pure electrospun PVDF-TrFE nanofiber and electrospun PVDF-TrFE with 1-3 wt% BTO nanoparticles; Typical output voltage signal of PVDF-TrFE (2 wt% BTO) sensor and PVDF-TrFE (3 wt% BTO) sensor at a fixed frequency of 7 Hz and various compressive forces; Output voltage parameters comparison of PVDF-TrFE (2 wt% BTO) and PVDF-TrFE (3 wt% BTO) under different pressures; Output voltage parameters of PVDF-TrFE (3 wt% BTO) under different pressures but at a constant frequency of 2 Hz; Output voltage parameters of PVDF-TrFE (3 wt% BTO) under different frequencies at a constant pressure of 4 N (6.4 kPa); Output voltage signal from devices with deposited Al and Al foil electrode at different frequencies but a fixed pressure of 2 N (3.2 kPa); Output voltage signal from finger pressing measured on an oscilloscope (PDF).

The pressing-generated voltage is wirelessly transmitted via Bluetooth to a smartphone for location detection. (MP4).

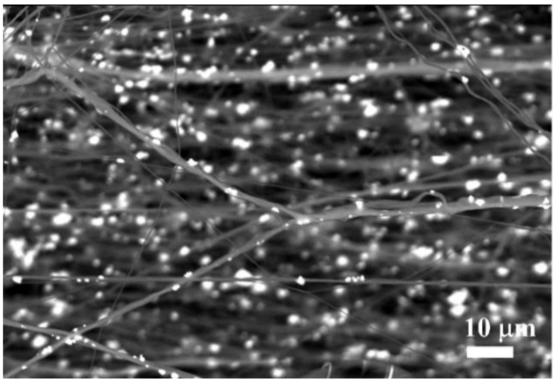

Figure S1. High-definition backscattered electron (HDBSD) SEM image of PVDF-TrFE (3 wt% BTO).

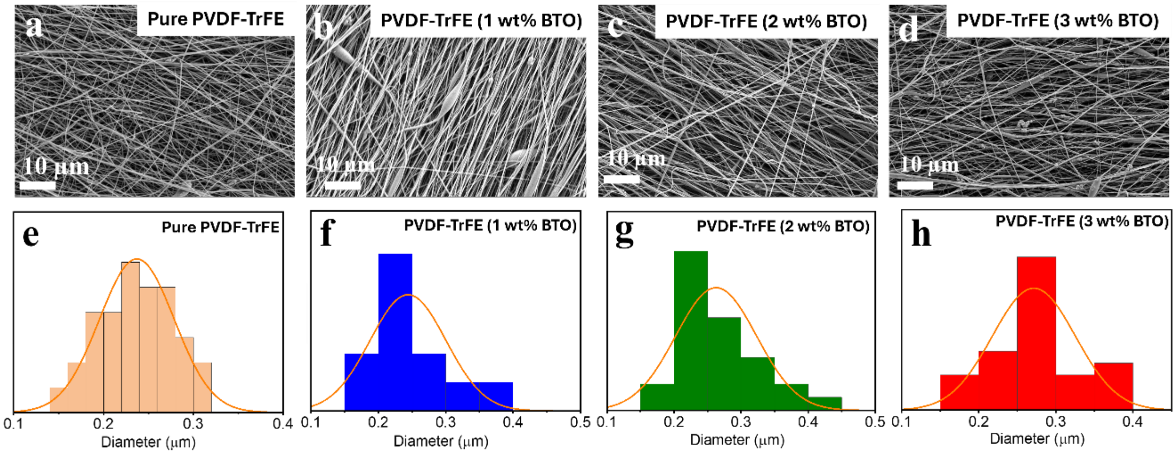

Figure S2. SEM images under low-magnification of (a) PVDF-TrFE, (b) PVDF-TrFE (1 wt% BTO), (c) PVDF-TrFE (2 wt% BTO) and (d) PVDF-TrFE (3 wt% BTO). The diameter distribution of (e) PVDF-TrFE, (f) PVDF-TrFE (1 wt% BTO), (g) PVDF-TrFE (2 wt% BTO) and (h) PVDF-TrFE (3 wt% BTO).

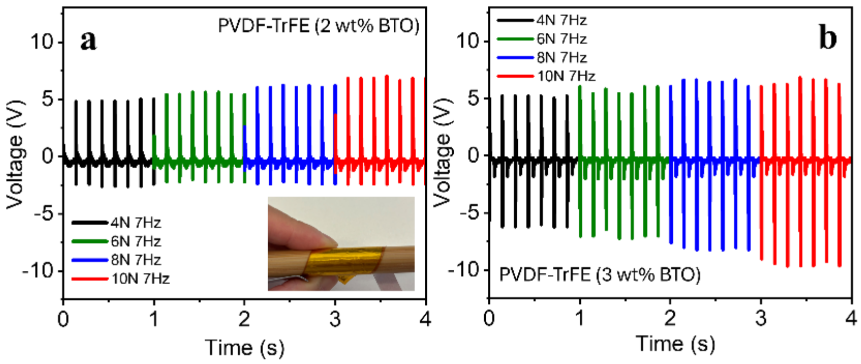

Figure S3. Typical output voltage signal of (a) PVDF-TrFE (2 wt% BTO) sensor and (b) PVDF-TrFE (3 wt% BTO) sensor under fixed frequency of 7 Hz and various compressive forces.

Table S1. Output voltage parameters of PVDF-TrFE (2 wt% BTO) under different pressures.

**Applied force/ Sample name**	**Compressive force (N)**	**4N 7Hz**	**6N 7Hz**	**8N 7Hz**	**10N 7Hz**
	Pressure (kPa)	**6.4**	**9.6**	**12.8**	**16.0**
**PVDF-TrFE (2 wt% BTO) Sample 1**	Positive Voltage (V)	5	5.6	6.2	7.0
	Negative Voltage (V)	-2.8	-2.4	-2.6	-2.6
	Average Voltage (V)	3.9	4.0	4.4	4.8
**PVDF-TrFE (2 wt% BTO) Sample 2**	Positive Voltage (V)	2.8	3.5	3.8	4.2
	Negative Voltage (V)	-2.0	-2.3	-2.5	-2.7
	Average Voltage (V)	2.4	2.9	3.15	3.45
**PVDF-TrFE (2 wt% BTO) Sample 3**	Positive Voltage (V)	2	2.7	3.3	3.7
	Negative Voltage (V)	-1.5	-1.8	-2.2	-2.7
	Average Voltage (V)	1.75	2.15	2.75	3.2

Table S2. Output voltage parameters of PVDF-TrFE (3 wt% BTO) under different pressures.

**Applied force/ Sample name**	**Compressive force (N)**	**4N 7Hz**	**6N 7Hz**	**8N 7Hz**	**10N 7Hz**
	Pressure (kPa)	**6.4**	**9.6**	**12.8**	**16.0**
**PVDF-TrFE (3 wt% BTO) Sample 1**	Positive Voltage (V)	5.4	6.2	6.8	7.0
	Negative Voltage (V)	-6.4	-7.2	-8.4	-9.8
	Average Voltage (V)	5.7	6.7	7.6	8.4
**PVDF-TrFE (3 wt% BTO) Sample 2**	Positive Voltage (V)	2.8	3.5	4.2	5.9
	Negative Voltage (V)	-3.0	-5.0	-7.4	-9.0
	Average Voltage (V)	2.9	4.25	5.8	7.45
**PVDF-TrFE (3 wt% BTO) Sample 3**	Positive Voltage (V)	5.4	7.5	8.9	10
	Negative Voltage (V)	-2.8	-3.5	-4.2	-5.4
	Average Voltage (V)	4.1	5.5	6.55	7.7

Table S3. Output voltage parameters of PVDF-TrFE (3 wt% BTO) under different pressures at a constant frequency of 2 Hz.

PVDF-TrFE-BTO_3	4N 2Hz	6N 2Hz	8N 2Hz	10N 2Hz	12N 2Hz	14N 2Hz
**Pressure (kPa)**	**6.4**	**9.6**	**12.8**	**16.0**	**19.2**	**22.4**
**Positive Voltage (V)**	3.2 V	4.0 V	4.6 V	4.8 V	5.8 V	6.2 V
**Negative Voltage (V)**	-1.0 V	-1.4 V	-3.4 V	-4.2 V	-5.8 V	-6.4 V
**Average voltage (V)**	2.1 V	2.7 V	4.0 V	4.5 V	5.8 V	6.3 V

Table S4. Output voltage parameters of PVDF-TrFE (3 wt% BTO) under different frequencies at a constant pressure of 4 N (6.4 kPa).

PVDF-TrFE-BTO_3	4N 4Hz	4N 5Hz	4N 6Hz	4N 7Hz
**Positive Voltage (V)**	4.2 V	4.8 V	5.2 V	5.4 V
**Negative Voltage (V)**	-4.0 V	-5.2 V	-5.6 V	-6.4 V
**Average (V)**	2.1 V	5.0 V	5.4 V	5.9 V

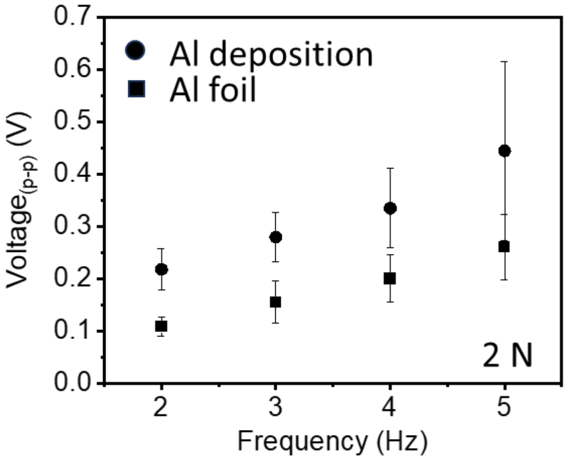

Figure S4. Output voltage signal from devices with deposited Al and Al foil electrode under different frequencies at a fixed pressure of 2 N (3.2 kPa).

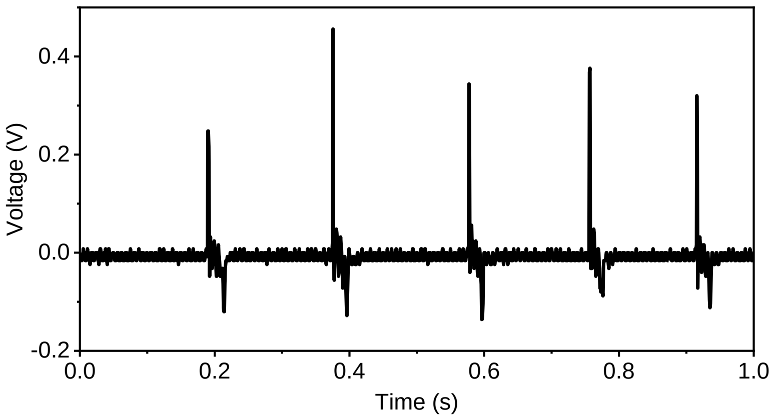

Figure S5. The output voltage signal from manual finger tapping is measured on an oscilloscope.
